# When a Fly Ball Is Out of Reach: Catchability Judgments Are Not Based on Optical Acceleration Cancelation

**DOI:** 10.3389/fpsyg.2017.00535

**Published:** 2017-04-07

**Authors:** Dees B. W. Postma, Joanne Smith, Gert-Jan Pepping, Steven van Andel, Frank T. J. M. Zaal

**Affiliations:** ^1^Center for Human Movement Sciences, University Medical Center Groningen, University of GroningenGroningen, Netherlands; ^2^School of Exercise Science, Faculty of Health Sciences, Australian Catholic University, BrisbaneQLD, Australia

**Keywords:** Chapman strategy, optical acceleration cancelation strategy, affordances, catchability, fly balls, baseball, interception, perception and action

## Abstract

The optical acceleration cancelation (OAC) strategy, based on Chapman’s (1968) analysis of the outfielder problem, has been the dominant account for the control of running to intercept fly balls approaching head on. According to the OAC strategy, outfielders will arrive at the interception location just in time to catch the ball when they keep optical acceleration zero. However, the affordance aspect of this task, that is, whether or not an approaching fly ball is *catchable*, is not part of this account. The present contribution examines whether the scope of the OAC strategy can be extended to also include the affordance aspect of running to catch a fly ball. This is done by considering a fielder’s action boundaries (i.e., maximum running velocity and –acceleration) in the context of the OAC strategy. From this, only when running velocity is maximal and optical acceleration is non-zero, a fielder would use OAC to perceive a fly ball as uncatchable. The present contribution puts this hypothesis to the test. Participants were required to try to intercept fly balls projected along their sagittal plane. Some fly balls were catchable whereas others were not. Participants were required to catch as many fly balls as possible and to call ‘no’ when they perceived a fly ball to be uncatchable. Participants’ running velocity and –acceleration at the moment of calling ‘no’ were examined. Results showed that participants’ running velocity was submaximal before or while calling ‘no’. Also running acceleration was often submaximal. These results cannot be explained by the use of OAC in judging catchability and ultimately call for a new strategy of locomotor control in running to catch a fly ball.

## Introduction

Making a catch in baseball is arguably one of the most spectacular ways to gain advantage on the batting team. Whenever a catch is made, the batter is out and the runners have to tag-up (i.e., touch their time-of-pitch base), providing the fielding team with a unique strategic advantage. Running to intercept a fly ball can be a demanding task in which careful locomotor control is required to get to the right place in the right time to make the catch. A large number of studies have addressed the locomotor control involved in catching fly balls. The strategy most widely accepted to account for the control of running to catch fly balls that approach head on is *the Chapman strategy* (Chapman, 1968; McBeath et al., 1995; McLeod et al., 2002, 2006, 2008; Shaffer and McBeath, 2002; Shaffer et al., 2003, 2008; Fink et al., 2009). Paradoxically, a hardly addressed issue in this account of catching fly balls is that of perceived catchability. Some fly balls are catchable whereas others are not. Catchability depends on the action boundaries of a fielder (e.g., maximum running velocity and –acceleration) as well as on the flight characteristics of a ball (e.g., flight time and –distance). That is to say, when the ball flight allows enough time for a fielder to cover the distance to the interception location, the ball would be catchable. If, however, the fielders’ running capabilities make that more time is needed to run to that same location, this would yield the ball uncatchable. Presumably, this affordance of catchability plays a role in running to catch a fly ball (Fajen, 2007). For example, if a fly ball is perceived uncatchable, a fielder’s primary goal might no longer be to make the catch, but to get the ball after the first bounce. The latter situation requires different timing and coordination, illustrating that perceived catchability could have a profound effect on the way locomotion is controlled. In addition, it might well be the case that fielders will speed up when their current running speed only just suffices to reach the interception location (just as shown to be the case in engaging the brake in driving; see Fajen, 2005a). In this study, we will examine whether the scope of the Chapman strategy, the dominant account for the timing of running to catch fly balls, can be extended to also include the affordance aspects of this task (i.e., perceived catchability). This will be a first step toward developing an affordance-based control account (Fajen, 2007) for catching fly balls.

Chapman (1968) considered the dynamics of running to catch a fly ball. Although Chapman did consider the forward–backward as well as the lateral component of running to catch fly balls, the former has received the most attention, probably because this component in his analysis determines the timing of interception. For a ball approaching an outfielder head on, he showed that the rate of change of the tangent of the elevation angle *α* of the ball [i.e., *d*(tan α)/dt] is constant for the constant running velocity that would lead a fielder to the right place in the right time to intercept a fly ball. Deviations from a constant value of *d(tan α)/dt* can be corrected for by adjusting locomotor velocity. An increase in *d(tan α)/dt* specifies that a fielder’s current locomotor velocity is such that the ball will fly overhead, whereas a decrease in *d(tan α)/dt* specifies that the fielder’s locomotor velocity is insufficient to reach the landing location in time. Adjusting locomotor velocity such that *d*(tan α)/dt remains constant has become known as the Chapman strategy (e.g., Chapman, 1968; Michaels and Oudejans, 1992; Dienes and McLeod, 1993; McLeod and Dienes, 1996; Michaels and Zaal, 2002; Zaal and Michaels, 2003; Kistemaker et al., 2008).

For an outfielder to take advantage of the Chapman strategy, information related to *d*(tan α)/dt must be optically available. That is to say, a fielder must be able to perceive whether his or her current locomotor efforts are sufficient for keeping constant *d*(tan α)/dt. When fielders track the ball with their gaze (see Oudejans et al., 1999; Postma et al., 2014), the elevation angle *α* equals the gaze angle. In this case, gaze angle can be the basis for using the Chapman strategy. A more general solution, also useful when gaze is not directed at the ball, can be found in the optical array (Todd, 1981; cf. Michaels and Oudejans, 1992). From a fielder’s point of view, the optical position of the ball—the projection of the ball on a planar projection plane—will rise at constant speed if *d*(tan α)/dt is constant. Thus, in essence, the Chapman strategy amounts to keeping optical velocity constant. Since keeping optical velocity constant is equivalent to nulling optical acceleration, the Chapman strategy is also known as the optical acceleration cancelation (OAC) strategy. If optical velocity is not constant (i.e., optical acceleration ≠ 0), *d*(tan α)/dt is not constant either and the fielder must make locomotor adjustments to make it in time to make the catch. For reasons of consistency, we will only use the term OAC strategy from here on out.

Empirical studies have shown that fielders’ locomotor patterns are consistent with the OAC strategy. For successful interception, fielders have been shown to run in such a way that optical acceleration equals zero for the largest part of their running movement (Michaels and Oudejans, 1992; Dienes and McLeod, 1993; McLeod and Dienes, 1996). Fink et al. (2009) consolidated this finding by showing that locomotor patterns were not merely coincidental to naturalistic interception of fly balls, but actually resulted from online visual control of optical acceleration. To test this, Fink et al. (2009) had participants intercept baseballs in virtual reality. This allowed the experimenters to perturb the trajectory of a ball mid-flight. Results showed that participants corrected for optical acceleration, resulting from perturbations to the ball trajectory, in order to make the catch. Not only locomotor behavior appears to be in line with the OAC strategy, also gaze behavior fits the use of optical acceleration. It has been shown that participants maintain continuous visual contact with the ball while running to make a catch, even for balls that fly overhead (Oudejans et al., 1999; Postma et al., 2014). Such gaze behavior fits naturally with the use of the OAC strategy, as it implies continuous visual control of interception.

The OAC strategy is in essence an error-nulling strategy (McBeath et al., 1995; Fajen, 2005a,b,c, 2007). Nulling optical acceleration, by adjusting locomotor velocity, will lead a fielder to the right place in the right time to make a catch. However, nulling optical acceleration is a condition that is not always possible to satisfy. The change in optical acceleration that a fielder can bring about by adjusting running velocity and/or -acceleration is limited by the locomotor abilities of the fielder. That is to say, locomotor constraints on part of the fielder, determine whether optical acceleration can be nulled. This is the problem of action boundaries (Fajen, 2005a,b,c, 2007). Action boundaries constrain what one can and cannot do: some fly balls are catchable while others are not. Fajen (2005a,b,c) argued that people are aware of their action boundaries and that they act accordingly to control behavior. As such, behavioral control strategies should not only be about sufficiency but also about possibility. What lies within the action boundaries of an actor to attain a certain goal? Or analogously: what is afforded given the action boundaries of the actor? Based on this notion, Fajen (2005a,b,c, 2007) developed the concept of *affordance-based control*: a novel conceptualization of motor control in which affordances, or action possibilities, rather than error-nulling principles are cardinal to understanding behavior (for an excellent review on affordance-based control, please refer to Fajen, 2007).

In the present contribution, we examined whether the OAC strategy can be made compatible with the concept of affordance-based control. While the OAC strategy is an error-nulling strategy and action boundaries have no part in its original formulation, optical acceleration might still be used in perceiving the affordance for catchability. From the OAC strategy, catchability can be observed under two specific conditions. First, if optical acceleration equals zero, and assuming that a fielder’s current locomotor velocity can be maintained, the fielder knows that a fly ball will be *catchable*. Second, a fielder knows that a fly ball will be *uncatchable* when optical acceleration does not equal zero but locomotor velocity cannot be further increased (either forward or backward) to cancel out optical acceleration. In this case, the locomotor qualities of the fielder serve as a constraint on optical acceleration cancelation, informing the fielder of (un)catchability. Thus, from the OAC strategy, catchability can be directly perceived from either one of these aforementioned conditions.

However, when optical acceleration does not equal zero *and* the fielder is not running at maximum speed, optical acceleration cannot inform about catchability. To appreciate why this is the case, it is important to understand that there is no one-to-one relationship between optical acceleration and the locomotor adjustment required to null the error. This stems from the fact that optical acceleration is determined by optical position, which is a function of the position of the fielder relative to the ball. Optical position follows one-to-one from a specific fielder-ball geometry. Yet, the reverse is not true: no specific fielder-ball geometry follows from optical position. To illustrate this, consider a fly ball approaching a fielder head on. At some point in time, the fly ball is positioned both 1 m above eye-level and 1 m in front of the fielder. From this specific fielder-ball geometry, it can be derived that the angle at which the ball approaches the fielder is 45°. Yet, solely from optical position (in this case 45°), it cannot be derived that the ball is 1 m in front of the fielder and 1 m above eye-level. The ball might as well be 10 m up and 10 m away (given that the optical size of the ball has no part in the OAC strategy). As such, optical position holds no information about distance, and hence there are an infinite number of ball positions that give rise to the same optical position. Consequently and analogously, the magnitude of optical acceleration holds no information about the required locomotor adjustment or whether this adjustment is within the action boundaries of the fielder. Ergo, the affordance of catchability cannot be directly perceived when the fielder is not running maximally *and* optical acceleration is non-zero. From the OAC strategy, catchability can thus only be perceived under specific circumstances.

In this study, we examined whether judgments of perceived catchability would fit the use of the OAC strategy. To test this, we designed an experiment in which participants were required to intercept tennis balls projected along their sagittal plane. Some balls were projected within the locomotor range of the participant (these were potentially catchable), whereas others were projected beyond the locomotor range of the participant (these were uncatchable). Participants were required to intercept as many fly balls as possible and were instructed to call ‘no’ whenever they felt that a fly ball would be uncatchable. The kinematic profiles for trials in which a participant called ‘no’ were studied to establish whether judgments of catchability could have been based on having reached maximum running velocity and/or maximum running acceleration, as would be expected from the OAC strategy.

## Materials and Methods

### Participants

Two women and four men, aged (20–27) volunteered to take part in the experiment. All participants had considerable experience in ball sports (at least 11 years), yet none of them had experience with baseball *per se*. All participants reported normal or corrected to normal vision. Prior to the experiment all participants were informed about the procedure and gave their written informed consent. The experiment was approved by the Ethics Board of the Center for Human Movement Sciences (University Medical Center Groningen, the Netherlands), and the protocol was in accordance with the Declaration of Helsinki.

### Design

Participants were required to intercept tennis balls projected at them along their sagittal plane. Tennis balls were projected either in front of- (front-trials) or behind the initial position of the participant (back-trials). Some fly balls were catchable (i.e., within the locomotor range of the participant) whereas others were uncatchable (i.e., beyond the locomotor range of the participant). Catchability was manipulated by systematically varying both flight time (1.64–3.00 s) and projection distance (10–20 m for front-trials and 20–30 m for back-trials), resulting in 24 ball trajectories (see below). We aimed at delivering an equal number of front- and back trials, and to have these sets equivalent in terms of flight time and passing distance (i.e., the distance between the initial position of the participant and the landing location of the ball). The initial position of the participant (20 m from the site of ball projection) was the same for all trials. Participants received a total of 96 trials, which were block-randomized over 4 blocks of 24 trials. Participants were encouraged to intercept as many fly balls as possible and were instructed to call ‘no’ at the moment that they realized a fly ball to be uncatchable. No specific instructions were given with regard to catching strategies (i.e., underhand- or overhand catching). At the start of each trial, the experimenter verbally cued the participant for ball delivery.

### Setup and Apparatus

The experiment was performed in a large gymnasium (50 m × 30 m × 10 m). Tennis balls were delivered using a pitching machine with adjustable pitch and power (Louisville Slugger, type UPM45 Blue Flame). To realize a range of projection angles beyond the factory settings of the pitching machine, it was mounted on a wooden board that could be placed at a particular angle using wooden blocks of different heights. The height of the blocks was such that the machine could be tilted 10–40°. We used 24 sets of pitch, power, and angle combinations in the experiment. Due to slight inherent variability of the apparatus, ball trajectories at identical apparatus settings were never exactly the same. As such, the projection distance could be manipulated with an approximate accuracy of 0.5 m. To prevent visual anticipation of the ball trajectory, the pitching machine was occluded from sight using a screen.

The experiment was recorded using an HD-camera (Canon HF100) positioned perpendicular to the plane of ball projection. The camera was set to its minimal focal length and a ×0.45 wide-angle converter was used to further increase the visual angle (122°). The camera was mounted on a tripod and a fast shutter speed (1/200) was used to prevent motion blur. The experiment was recorded at a frame rate of 25 frames per second.

### Data Analysis

Data from the HD-camera was imported and converted to *.MOV files using QuickTime Player (v. 10.3). Video-files were trimmed down to individual trials. The first frame associated with ball projection constituted the start of a trial. The end of a trial was dependent on its outcome (i.e., success or failure). Trials in which the participant managed to catch or touch the ball (i.e., success) were digitized up until the moment of first contact, whereas trials in which the participant failed to catch, or even touch, the ball (i.e., failure) were digitized up until the moment the ball hit the floor. Finally, Audacity (v. 1.2.6) was used to determine if and when a participant called ‘no’.

The planar coordinates of the ball and the participant’s head were digitized on a frame-to-frame basis (NBody, v.09-13; E. Otten). Using a planar checkerboard pattern, lens distortion was calculated and corrected for. The position of the ball was retrieved by identifying differences between subsequent frames through subtraction of RGB-values on pixel-level. Differences between frames were highlighted after all the frames of a trial were analyzed. From these highlighted regions, the trajectory of the ball was manually specified. The head position of the participant was digitized using a custom-made shape recognition algorithm. Whenever the position of the participant’s head could not be established automatically the position of the participant’s head was digitized manually. The digital coordinates of both the ball and the participant were transformed to real world metrics using a quaternion. The site of ball projection constituted the origin of the quaternion with the x-axis extending toward the participant and the y-axis extending toward the ceiling. The data were filtered and smoothed for final analysis. A fourth order polynomial function was used to account for missing values in the ball data; a cubic spline was used to interpolate and filter the participant data (smoothing parameter: 0.995).

To assess the running velocity and –acceleration at the moment of calling ‘no’, kinematic profiles were calculated by differentiating the participant’s positional data. Subsequently, kinematic profiles were transformed such that positive values constituted motion in the direction required to make a catch. This allowed for direct comparison of running characteristics of front-trials and back-trials. Additionally, running velocity and –acceleration at the time of calling ‘no’ were expressed as a percentage of participants’ maximum running velocity and –acceleration. For each participant, maximum running velocity and –acceleration were operationally defined as the highest values for running velocity and –acceleration over all trials for that participant (corrected for passing side). Finally, all values were summarized using probability density plots.

## Results

Out of a total of 576 trials, 27 trials were excluded because the ball hit the ceiling. Furthermore, 36 trials could not be digitized due to technical difficulties (e.g., irregularities in the background causing erratic tracking behavior). From the remainder of trials, only those in which a participant called ‘no’ were selected for further analysis (*n* = 218). For these trials, the position of the ball could be established in 68.7% of all frames, while the position of the participant could be established in 98.4% of all frames. Missing values were accounted for as detailed above: for the ball data a fourth order polynomial spline was used whereas a cubic spline was used for the participant data. No participants were excluded from further analysis.

On average, participants caught 50.2% (*SD* = 9.7%) of the balls projected at them. Conversely, on average, participants judged 39.8% (*SD* = 12.9%) of the fly balls projected at them to be uncatchable. On a small number of trials (*M* = 5.4%, *SD* = 3.7%) participants failed to intercept the ball while not calling ‘no’ either. In the remainder of the trials, the ball hit the ceiling (as detailed above). It should be noted that participants never called ‘no’ for fly balls that were eventually caught.

**FIGURE 1:**
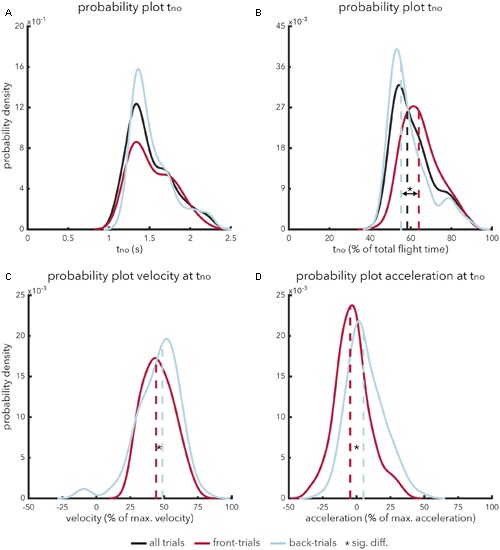
**(A–D)** Probability density plots showing the relative likelihood of relevant temporal-spatial characteristics in indicating that a fly ball is perceived to be uncatchable. The abscissa represents the range of relevant values associated with the variable in question. The ordinate represents probability density values. The integral of a specific range provides the cumulative probability of value *x* of property *y* falling within that range. The integral of a probability density function is always equal to 1. Probability functions (solid lines) are presented along with a graphical representation of the mean (dashed lines) for front-trials (red lines), back-trials (blue lines) and, if applicable, all trials (black lines). **(A)** Represents the probability density function for the absolute timing of calling ‘no’ from trial onset. **(B)** Represents the probability density function for the relative timing of calling ‘no’; i.e., expressed as a percentage of the total duration of a trial. **(C)** Represents the probability density function for the running velocity when calling ‘no’, expressed as a percentage of maximum velocity. Finally, **(D)** represents the probability density function for running acceleration when calling ‘no’, expressed as a percentage of maximum acceleration.

The temporal pattern of calling ‘no’ (*t*_no_) followed a bell-shaped distribution (**Figure 1A**). Participants never took less than 0.8 s to indicate that a fly ball was uncatchable. On average, it took participants 1.42 s (*SD* = 0.40 s) to indicate that a fly ball was judged to be uncatchable. An independent-samples *t*-test showed that there was no significant difference [*t*_(216)_ = -0.70, *p* = 0.482, *d*_s_ = -0.97] in absolute timing of calling ‘no’ for front-trials (*M* = 1.41 s, *SD* = 0.45 s) as compared to back-trials (*M* = 1.45 s, *SD* = 0.36 s). In contrast, a significant effect was apparent [*t*_(216)_ = 4.27, *p* < 0.001, *d_s_* = 0.59] when the timing of calling ‘no’ (*t = no*) was expressed as a percentage of total flight time (**Figure 1B**). Participants judged fly balls to be uncatchable earlier in ball flight for back-trials (*M* = 55.5%; *SD* = 14.1%) as compared to front-trials (*M* = 63.8%; *SD* = 13.9%).

To assess the usefulness of optical acceleration in terms of catchability, we will turn to the kinematics of the participants. As discussed before, taking the OAC strategy as framework, catchability can be judged under one of two specific circumstances: either optical acceleration equals zero, indicating that a ball is catchable, or optical acceleration does not equal zero *and* the participant is running at his or her maximum velocity, indicating that a fly ball is uncatchable. To test whether perceived catchability fits the OAC strategy, we analyzed participants’ running velocity (and –acceleration) at the time of calling ‘no’ (*t_no_*). The results show that participants never ran at their maximum velocity at *t_no_* (**Figure 1C**). In fact, on occasions, participants were even standing still while calling ‘no’. On average, participants ran at 46.4% (*SD* = 7.9%) of their maximum speed when calling ‘no’. An independent-samples *t*-test showed that participants exhibited a significantly [*t*_(216)_ = -2.14, *p* = 0.034, *d_s_* = -0.30] lower relative running speed while calling ‘no’ in front-trials (*M* = 43.3%; *SD* = 19.1%) as compared to back-trials (*M* = 49.4%; *SD* = 21.8%), see also **Figure 1C**. Interestingly, for back-trials, participants occasionally called ‘no’ while running in the wrong direction (i.e., toward the site of ball projection), as can be seen from the small negative peak in **Figure 1C**.

Rather than maximum running velocity, participants might also have used maximum acceleration in making perceptual judgments of catchability. However, the results show that participants also never accelerated maximally at *t_no_*. Participants were on average decelerating at the moment of calling ‘no’ in front-trials (*M* = -4.7%; *SD* = 18.1%), whereas participants were on average accelerating in back-trials (*M* = 4.94%; *SD* = 19.2%). Using an independent-samples *t*-test, this difference was found to be significant [*t*_(216)_ = -3.50, *p* < 0.001, *d_s_* = -0.48], see also **Figure 1D** (it is important to note that the kinematic profiles were transformed such that a positive value for either velocity or acceleration constitutes motion in the required direction).

Finally, we examined the peak locomotor values (i.e., maximum velocity and –acceleration) reached by participants before calling ‘no’. On average, participants reached a peak velocity of 54.0% of their maximum running velocity (*SD* = 19.1%) and a peak acceleration of 71.2% of their maximum running acceleration (*SD* = 15.6%) before calling ‘no’. Participants rarely reached peak locomotor values greater than 90% of their maximum running velocity (*n* = 2) or –acceleration (*n* = 19), before calling ‘no’ (see also Supplementary Figure 1). These trials made out only 0.9% and 8.7% respectively of all trials in which a participant called ‘no’.

## Discussion

Making a catch in baseball provides the fielding team with a unique strategic advantage. In the 1954 World Series, Willie Mays turned the odds in his favor by successfully intercepting Vic Wertz’ towering smash. When interviewed about his performance, Mays stated: *“I knew I had the ball all the time”* (Willie Mays Interview – Academy of Achievement, 2017)^1^. As such, this memorable play (known as ‘the catch’) was contingent on Mays’ perception of the affordance of catchability. In this study, we assessed whether the dominant account for the control of the forward–backward component of running to catch a fly ball (i.e., the OAC strategy) can be extended to include the affordance aspects of intercepting fly balls. Note that several accounts are available for the lateral component of running to catch fly balls, and the coupling of the lateral and forward–backward components (Chapman, 1968; McBeath et al., 1995; McLeod et al., 2001, 2006; Shaffer et al., 2003; Fink et al., 2009). Here, we restrict ourselves to the control of forward–backward running.

Chapman (1968) proposed that the rate of change of the tangent of the elevation angle of the ball is constant for the constant running velocity that would lead a fielder to the right place in the right time. Depending on the distance a fielder has to cover and the time that is available to do so, this constant running velocity can take on any value. Running velocity can be really low for fly balls that are easy to catch and really high for fly balls that are almost impossible to catch. Yet, in its origin, the OAC strategy provides no means for separating catchable from uncatchable fly balls, in part because there is no one-to-one relation from optical acceleration values to catchability. We reasoned, however, that the OAC strategy could still work for perceiving catchability if fielders’ action boundaries are taken into account (Fajen, 2005a,b,c, 2007; Harrison et al., 2016). From this, the OAC strategy could still specify the affordance of catchability under specific circumstances. Either optical acceleration equals zero, indicating that a fielder will arrive at the right place in the right time to make a catch (i.e., the ball is catchable), or optical acceleration does not equal zero while a fielder is running to the best of his/her abilities, indicating that a fly ball is uncatchable. We found, however, that participants’ judgments of uncatchable fly balls were not confined to these particular circumstances. In fact, participants rarely ran at maximum velocity or –acceleration while judging a fly ball to be uncatchable.

Whenever, participants called ‘no’ their running velocity was often far from maximal; indeed participants could even be standing still while doing so. The same goes for running acceleration. Participants were hardly accelerating, or even decelerating, as they called ‘no’. These findings contrast the aforementioned prerequisites for perceiving the affordance of catchability from the use of optical acceleration. One might argue, however, that the decision to call ‘no’ is not reflective of the instant that a participant *actually* perceived a fly ball to be uncatchable. As such, perceptual judgments might have resulted from participants already having reached their locomotor maximum at an earlier moment. Yet, examination of the kinematic profiles revealed that this was not the case either: participants hardly ever reached their maximum running velocity or –acceleration before calling ‘no’. Note that the maximum velocities and accelerations as determined for every participant might actually be underestimated. That is to say, we took their maximum values in the experiment, and it might well be possible that their actual maximum values were higher when allowed to run longer distances. Still, even so, the argument that participants did not reach their maximum running velocity or –acceleration before calling ‘no’ holds. These findings suggest that it is unnecessary for fielders to exert, or to have exerted, maximum locomotor effort when judging the catchability of a fly ball.

Knowing what one can and cannot do is essential to control of any type of behavior, including catching fly balls (Fajen, 2005a,b,c, 2007; Harrison et al., 2016). Fajen formalized the concept of affordance-based control with the task of braking a car to a safe stop (Fajen, 2005a,b,c, 2007), arguing that affordance aspects of situations are part of the control of action. In this study, we advocate affordance-based control and aimed to extend its principles to the fly ball paradigm. Having established that the OAC strategy cannot be easily extended to include the affordance aspects of fly ball catching can be seen as a first step in doing so. One complication in making the next steps in developing the affordance-based account is that it is not clear how to characterize the affordance of catchability. One reason that using the OAC strategy did not work out very well was that this strategy yields no information on when an approaching fly ball would be landing where. This means that the task of identifying the affordance (i.e., the relation between ball-flight characteristics and player abilities) is not straightforward from a scientific point of view. A next step in the hunt for an affordance-based account, thus, might be an attempt to lay out the variables that combine to classify some fly balls as catchable and others as uncatchable. For present purposes, we have worked from the assumption that the ‘no’-s of our participants were sufficiently accurate. Understanding how ball-trajectory characteristics combined with player abilities to yield the affordance of catchability would allow us to check this assumption. More importantly, however, it would possibly guide us to uncovering the information that people use to perceive the affordance of catchability of approaching fly balls. Finally, the temporal patterns that we observed when participants gave their ‘no’-s might prove useful to arrive at a more precise characterization of the optical variables that they used. Potentially, the optics at the moment that (or just before) the moment the participants called ‘no’ can be used to identify the optical variable. For certain ball trajectories participants were quick to respond, for others they needed more time. Because we are able to determine time series of positions of ball and head, time series of optical variables can be computed. Scrutinizing the latter time series around the moments of calling ‘no’ might prove to be the way to uncovering the optical variable that is at the basis of knowing when a ball is not catchable.

All in all, although the OAC- or Chapman strategy, provides a parsimonious (and, as such, is the dominant) account for locomotor control in catching fly balls, it does not appear to be easily extended to include the affordance aspects of catching a fly ball. Although our analyses showed this to be necessary for the OAC strategy to deal with catchability, neither running velocity nor –acceleration was maximal whilst judging a fly ball to be uncatchable. As such, the OAC strategy appears to be unable to explain how the perception of catchability comes to be.

## Author Contributions

The authors of this article hereby declare that they all had a significant and relevant part in the creation of this article. FZ, G-JP, and JS conceived and designed the experiment. Data acquisition was performed by JS and FZ. Data analysis was performed by DP, FZ, JS, and SvA. All authors were involved in interpreting the data. DP and FZ wrote the first drafts. Finally, all authors helped revising the article from draft to final version. All authors agree to be accountable for all aspects of the work in ensuring that questions related to the accuracy or integrity of any part of the work are appropriately investigated and resolved.

## Conflict of Interest Statement

The authors declare that the research was conducted in the absence of any commercial or financial relationships that could be construed as a potential conflict of interest.

## Footnotes

1 http://www.achievement.org/achiever/willie-mays/#interview
