# Supplementary figures and images for: When a Fly Ball Is Out of Reach: Catchability Judgments Are Not Based on Optical Acceleration Cancelation
Source: Front Psychol. 2017 Apr 7;8:535. doi: 10.3389/fpsyg.2017.00535 (PMC5383721; doi:10.3389/fpsyg.2017.00535)

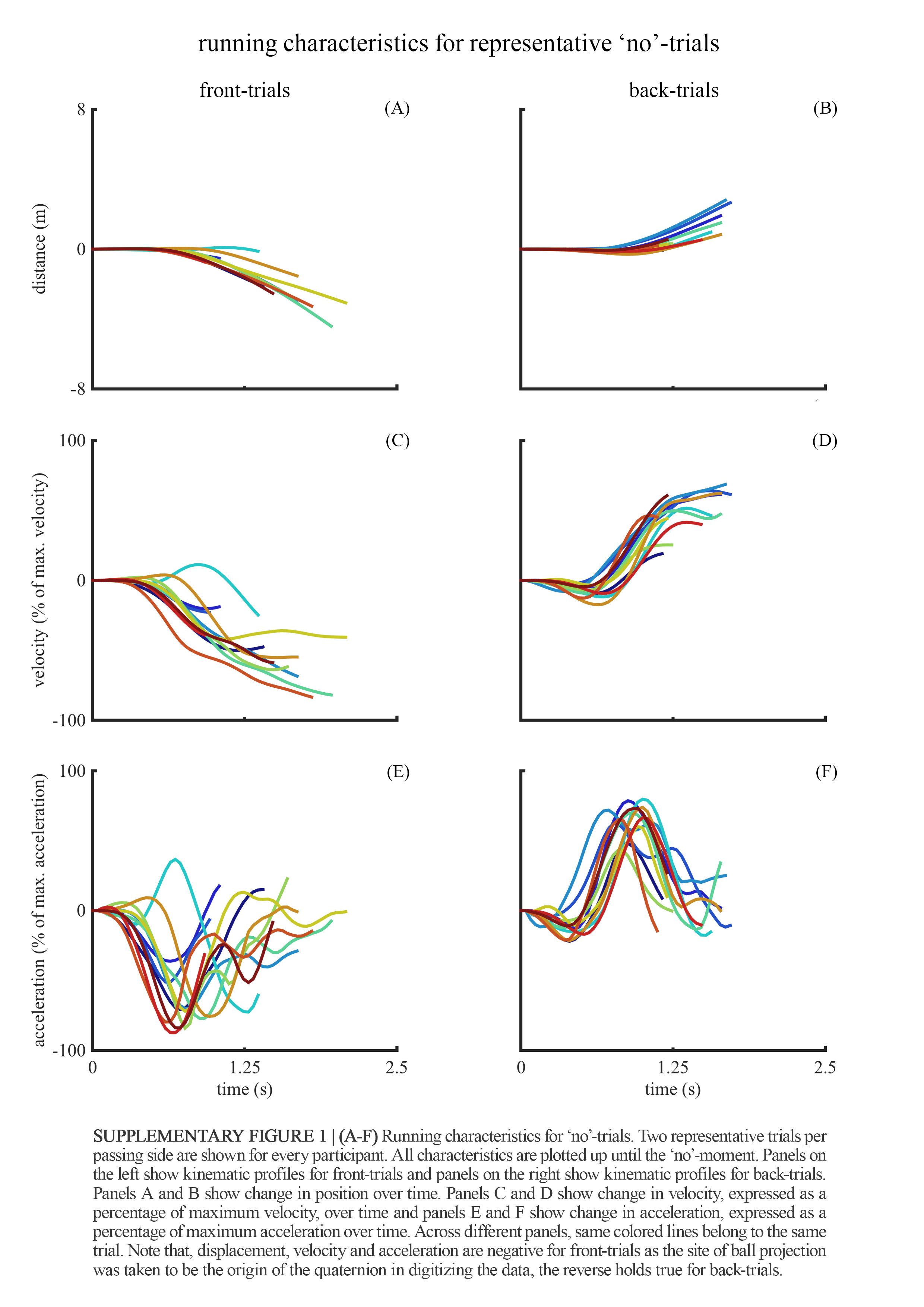

Supplement: Supplementary file 1 [file Image_1.tif]
